# Rapid assessment and prediction of the efficiency of two preservatives against *S*. *aureus* in cosmetic products using High Content Screening—Confocal Laser Scanning Microscopy

**DOI:** 10.1371/journal.pone.0236059

**Published:** 2020-07-27

**Authors:** Samia Almoughrabie, Chrisse Ngari, Laurent Guillier, Romain Briandet, Valérie Poulet, Florence Dubois-Brissonnet

**Affiliations:** 1 Micalis Institute, Université Paris-Saclay, INRAE, AgroParisTech, Jouy-en-Josas, France; 2 Laboratoires Clarins, Pontoise, France; 3 Direction de l’évaluation des risques, ANSES, Agence nationale de sécurité de l’alimentation, de l’environnement et du travail, Maisons-Alfort, France; University of Hong Kong, HONG KONG

## Abstract

Most cosmetic products are susceptible to microbiological spoilage due to contaminations that could happen during fabrication or by consumer’s repetitive manipulation. The composition of cosmetic products must guarantee efficient bacterial inactivation all along with the product shelf life, which is usually assessed by challenge-tests. A challenge-test consists in inoculating specific bacteria, i.e. *Staphylococcus aureus*, in the formula and then investigating the bacterial log reduction over time. The main limitation of this method is relative to the time-consuming protocol, where 30 days are needed to obtain results. In this study, we have proposed a rapid alternative method coupling High Content Screening—Confocal Laser Scanning Microscopy (HCS-CLSM), image analysis and modeling. It consists in acquiring real-time *S*. *aureus* inactivation kinetics on short-time periods (typically 4h) and in predicting the efficiency of preservatives on longer scale periods (up to 7 days). The action of two preservatives, chlorphenesin and benzyl alcohol, was evaluated against *S*. *aureus* at several concentrations in a cosmetic matrix. From these datasets, we compared two secondary models to determine the logarithm reduction time (Dc) for each preservative concentration. Afterwards, we used two primary inactivation models to predict log reductions for up to 7 days and we compared them to observed log reductions. The IQ model better fits datasets and the Q value gives information about the matrix level of interference.

## Introduction

Each year around the world, official authorities in Europe (Rapid Alert System for Non-Food Products) or USA (US Consumer Product Safety Commission) notify many recalls for cosmetic products due to microbiological contamination [1–3]. Cosmetic formulas are complex and are susceptible to microbiological spoilage due to their composition, containing water and nutrients such as lipids, polysaccharides, proteins [4]. Contamination of cosmetic products could happen during their fabrication but also by consumer’s repetitive manipulations [5, 6]. The main pathogens frequently found in cosmetic formulas are *Pseudomonas aeruginosa*, *Escherichia coli*, *Burkholderia cepacia*, *Candida albicans*, *Klebsiella oxytoca*, *Enterobacter gergoviae*, *Serratia marcescens and Staphylococcus aureus* [2, 7–9]. *S*. *aureus* has been found in various cosmetic products such as shaving cream, moisturizing cream, face care cream and depilatory cream [10–12]. It is a Gram-positive bacterium present on human skin and mucous membranes in 30% of the population [13]. Many *S*. *aureus* strains produce exfoliative toxins secreted on the skin that cause a wide range of clinical infections, including abscesses, furuncles or impetigo [14–17].

Each cosmetic product has a different level of microbiological risk according to the standard ISO 29621:2017, which depends on several parameters such as the formula composition (preservative, ethanol, A_w_, pH) or the type of packaging (unidose, airless pump, pots) [6, 18]. Preservatives that can be used in cosmetic products are listed in Annex V of the European Regulation No. 1223/2009. Among them are listed chlorphenesin and benzyl alcohol, which have been tested in this study. Chlorphenesin or 3-(4-chlorophenoxy)-1,2-propanediol is an antifungal and antibacterial agent (active against both Gram-positive and Gram-negative bacteria). It can be used at a maximum concentration of 0.32% in rinse-off products and up to 0.30% in leave-on products [19]. Benzyl alcohol can be used in various cosmetic formulations as a preservative, but also as a solvent, a fragrance or a viscosity-controlling agent. Its maximum in-use concentration is 1% [20].

The preservation efficiency of a given product is evaluated by proceeding to a challenge-test, as defined in the European standard EN ISO 11930:2019. During this procedure, specific microorganisms, including *S*. *aureus*, are inoculated in the product at a final concentration between 1.10^5^ and 1.10^6^ CFU.ml^-1^ for bacteria, and 1.10^4^ and 1.10^5^ CFU.ml^-1^ for molds or yeast. The microbial population is evaluated at defined time intervals by enumerating the survivors at 7, 14 and 28 days after inoculation. A preservative system is considered as efficient against bacteria if the formula composition leads to a bacterial logarithm reduction ≥ 3 seven days after inoculation and without the growth of bacteria after 14 and 28 days. Challenge-test, as described in the European standard involves several steps including sampling, neutralization, serial dilutions, bacterial plating in duplicate, incubation time and colony counting [21]. The reliability of challenge-tests depends on several parameters such as the manipulation errors (pipetting and serial dilutions) [22], the type of plating method (spiral or pour plating), the level of bacterial enumeration [23], and on the ability of stressed microorganisms to recover and grow on agar plates [24]. It also relies on the efficiency of the neutralization step which consists of stopping the antimicrobial activity of preservatives by diluting the surviving population in a quenching solution [25]. The main limitation of the challenge-test procedure is relative to the time-consuming protocol (inoculation, sampling, counting) and to the duration of the whole test process (last sample analyzed on day 28).

Confocal Laser Scanning Microscopy (CLSM) allows *in-situ* 3-D visualization of microbial consortia thanks to various fluorescent markers. It is commonly used to investigate complex microbial spatial organizations such as biofilms [26], to analyze interactions between bacteria and oil droplets [27] or to evaluate bacterial distribution in food systems [28–30]. Moreover, CLSM was previously used to study the spatiotemporal action of biocide in biofilms [26, 31–34]. This method enables a real-time and *in situ* visualization of the bacterial inactivation kinetics after biocide addition. Typically, living cells are stained with an esterasic viability marker, such as cFDA or calcein-AM, and after subsequent biocide addition, the fluorescence is lost due to the leakage of the fluorescent marker out of the cell when the cell membrane is permeabilized.

In this study, we used CLSM and image analysis for acquiring datasets of bacterial inactivation kinetics upon short periods in model cosmetic matrices containing various concentrations of preservatives and we accurately predicted the number of bacterial log reductions on longer periods, which are similar to challenge-test ones.

## Materials and methods

### Chemicals and materials

Cetearyl glucoside and glyceryl stearate were purchased from SEPPIC (Puteaux, France), carbomer from Gattefossé (Lyon, France), glycerin from Oleon (Ertvelde, Belgium), cetearyl isononanoate from BASF France (Lyon, France), tocopheryl acetate from DSM (Heerlen, the Netherlands), tromethamine from Azelis (Heusden, Belgium), chlorphenesin and benzyl alcohol from Thor (Compiegne, France). Eugon LT 100 supplemented broth was purchased from Indicia production (Saint Genis l’Argentière, France).

### Bacterial strain and culture conditions

The strain used in this study is *Staphylococcus aureus* CIP 4.83 recommended by the EN ISO 11930:2019 standard for cosmetic-product challenge tests. It was stored in cryovials at -80°C and resuscitated by two successive subcultures in tryptic soy broth (TSB, Biomérieux, Marcy-l’étoile, France) before each experiment. Cultures were grown at 30°C until the end of the exponential growth phase.

### Preparation and characterization of the emulsified model matrix

The aqueous phase was first prepared with 0.25% carbomer in water and heated to 75°C before glycerin (moisturizer, 9%) was added. The oil phase is composed of 28.8% cetearyl isononanoate (emollient), 3.5% cetearyl glucoside (emulsifier), 0.2% tocopheryl acetate (antioxidant), and 2.5% glyceryl stearate (co-emulsifier). It was heated to 75–80°C before it was blended with the aqueous phase (20/80 o/w%) at 1,800 rpm using a rotor-stator homogenizer (Rayneri 33/300P, Group VMI) to obtain an emulsion. Benzyl alcohol and chlorphenesin at 7 different concentrations (respectively from 1.00 to 1.85% and from 0.30 to 0.60%) were respectively pre-mixed with glycerin or water at 40°C. Tromethamine (base, 0.15%) was finally added. The viscosity was measured using a penetrometer (PNR10, PetroMesures). A specific cone was released in 300 g of matrix and the penetration depth measured (in mm ± 0.1 mm) after 5 s. The penetrometry measured on each batch in triplicate is 33.06 mm ± 1.33 mm. The pH, measured using pH-meter (SI Analytics, Lab 870) on each batch in triplicate, is 5.76 ± 0.03.

### Bacterial staining and matrix inoculation

Bacterial cells were harvested by centrifugation at 1,575 *g* for 10 min and washed twice in 150 mM NaCl. The bacterial suspension was calibrated to 1.10^10^ to 1.10^11^ CFU.ml^-1^ in 150 mM NaCl to observe at least 10–100 bacteria per CLSM image (290.6 x 290.6 x 1.6 μm^3^). 300μl of bacterial suspension were labeled with 13 μl calcein-AM (53.55μM in DMSO, Invitrogen by Thermofisher Scientific), incubated in the dark for 1h30 at 37°C and inoculated in 30 g of model cosmetic matrix which was vortexed for 30 s. The average of the bacterial concentration in the matrix is 1.10^8^ to 1.10^9^ bacteria/g. Calcein-AM is a viability marker that penetrates passively into a cell where it is cleaved by cytoplasmic esterases and leads to green fluorescence. Each experiment was performed respectively two or three times from independent cultures for benzyl alcohol and chlorphenesin.

### Enumeration of the bacterial population by drop-plate method

For each enumeration, 1 g of inoculated matrix was dispersed in 9 ml of neutralization solution (Eugon LT 100 supplemented broth). After 30 minutes, the bacterial population is enumerated by serial dilution in 150 mM NaCl on tryptone soya agar (TSA, Biomérieux) using the drop-plate method [35]. Plates were incubated at 30°C for 24 to 48 h before counting. Bacterial enumeration is processed every twenty minutes for four hours after inoculation and then at least once every day until seven days. Each enumeration was performed at least in duplicate.

### Acquisition of bacterial inactivation curves by High Content Screening—Confocal Laser Scanning Microscopy (HCS-CLSM)

The evolution of bacterial population was acquired upon a short time (typically 4h) for 7 different concentrations in duplicate for benzyl alcohol and in triplicate for chlorphenesin. To obtain one inactivation curve, the inoculated matrix containing a specific concentration of a preservative was dropped into several wells of polystyrene 96-well microtiter plates (Greiner Bio-One, France) and CLSM acquisition was achieved in each well at a specific time to avoid photobleaching. Thanks to the HCS-CLSM, the stage was programmed to move automatically to the next well every 15 min during 4h or every hour during 13h for low concentrations.

Image acquisition was performed using a Leica SP8 AOBS Confocal Laser Scanning Microscope (Leica Microsystems, France) at the MIMA2 imaging platform (https://doi.org/10.15454/1.5572348210007727E12). Calcein-AM is excited at 488 nm and the emitted fluorescence collected in the range 498 to 560 nm. Images size were 290.6 x 290.6 x 1.6 μm^3^ (512 x 512 pixels) and were acquired at 600 Hz using a 40x air objective (N.A. = 0.85) and a hybrid detector. The HCS-CLSM control software was programmed to take a mosaic of 10 x 10 images per well, corresponding to a volume of 1.3 x 10^−5^ ml. The number of bacteria by mosaic was counted by binarizing each image using the MaxEntropy algorithm in an automatic macro executed in ImageJ software (National Institutes of Health, USA) [36]. The obtained number of bacteria per ml was converted per g according to the matrix density (1.15 g/ml). In our experimental conditions, we consider that our threshold value is at 1 bacteria per image or 100 bacteria per mosaic, which corresponds to 6.10^6^ bacteria/g.

### Primary model for bacterial inactivation on short times

The log-linear model of Bigelow *et al*. [37], described in Eq 1, was used to fit each CLSM inactivation curve acquired on short-times.

$${log}_{10}(N)\;=\;{log}_{10}(N_{0})-\frac{t}{Dc}$$ (1)

*where N*_*0*_ is the initial bacterial population, *N* is the bacterial population at the sampling time, *Dc* is the decimal reduction time and *t* is the time (min).

The GinaFit freeware add-in for Microsoft Excel was used to fit each curve [38] and to obtain the Dc value to which we applied a correction factor to take into account to the correlation between CLSM enumeration and plate enumeration. Hence, we obtained a dataset of Dc, each of them corresponding to a specific concentration of one preservative.

### Secondary model for estimation of the Dc-value according to the concentration

From obtained Dc datasets, the Dc values were fitted according to concentration using a semi-log approach, derived from Mafart *et al*. (2001) [39], and expressed in Eq 2.

$${log}_{10}(Dc)\;=\;{log}_{10}({Dc}^{*})-\;{\left(\frac{C-C^{*}}{z_{c}}\right)}^{n}$$ (2)

where *Dc* is the decimal reduction time for the concentration *C*, Dc* is the decimal reduction time for the reference concentration C*, z_c_ is the increase of concentration which leads to a ten-fold reduction of the decimal reduction, *n* is a shape parameter which can be set to 1 (model #1, linear model) or 2 (model #2, second-degree model). Dc* and z_c_ were the estimated parameters.

The model parameters were fitted with nls R function according to the minimization of the residual sum of square errors (RSS). Confidence intervals of fitted parameters were assessed by bootstrap using nlsBoot function from nlsMicrobio R package [40]. The two models were compared according to the Bayesian information criterion (BIC) (Eq 3). The lower the BIC, the better the model fits the dataset.

$$BIC\;=\;p.Ln(\frac{RSS}{p})+k.Ln(p)$$ (3)

Where *p* is the number of experimental points and k the number of parameters of the model.

### Prediction of the log-reduction of the bacterial population over a period of several days

To predict the log reduction of the bacterial population over several days, we first predict Dc with the secondary model #2 (Eq 2) at some tested concentrations of preservative.

Afterwards, two different models were used to predict the inactivation of the bacterial population as a function of time: the log-linear model (Eq 1) and IQ model (Eq 4). The intrinsic quenching model (Lambert et *al*., 2000) [41] was constructed with the hypothesis that the disinfection concentration decreases during the test period and can be described by the Eq 4.

$${log}_{10}(N)\;=\;{log}_{10}(N_{0})-\frac{\left(1-e^{-Q.t}\right)}{Q.D_{c}}$$ (4)

*where N*_*0*_ is the initial bacterial population, *N* is the bacterial population at the sampling time, *Dc* is the decimal reduction time, *t* is the time (min) and Q is the quenching coefficient. Q was the estimated parameter.

The logarithm reduction of the bacterial population that should be obtained after a defined time, from 1 to 7 days was predicted. To optimize and validate the model, a dataset of log-reductions of the bacterial population was acquired by plate enumeration on the corresponding periods (1 to 7 days) for 7 concentrations of each preservative, as described before. Predicted and observed log reductions were compared.

The model parameters were fitted with nls R function according to the minimization of the residual sum of square errors (RSS). Confidence intervals of fitted parameters were assessed by bootstrap using nlsBoot function from nlsMicrobio R package [40].

## Results

### Correlation between enumeration by CLSM and plate counting

**Fig 1:**
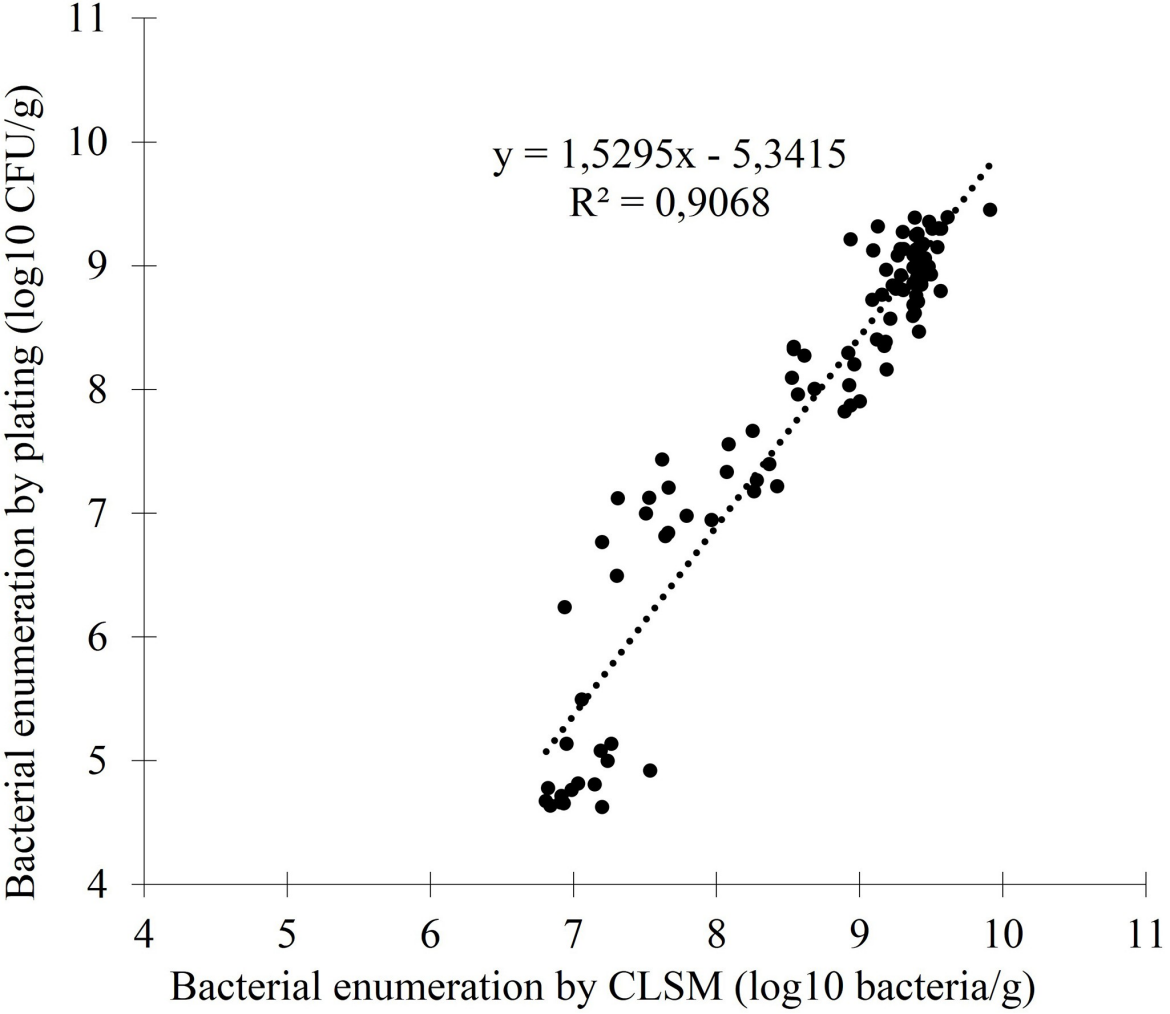
Correlation between bacterial enumeration by plating (log_10_ CFU/g) and bacterial enumeration by CLSM imaging (log_10_ bacteria/g).

Model cosmetic matrices were formulated with different concentrations of chlorphenesin or benzyl alcohol. Bacterial enumeration of *S*. *aureus* was achieved at several contact times (between 10 min and 4h) by both plate counting (log CFU/g) and CLSM enumeration (log bacteria/g). Fig 1 gives the relationship between both techniques. The relationship between both techniques is linear (y = 1.530x – 5.342; R^2^ = 0.907) for a level of population over the detection threshold of the technique (6.10^6^ bacteria/g).

### Inactivation of *S*. *aureus* according to the concentration of the preservative

**Fig 2:**
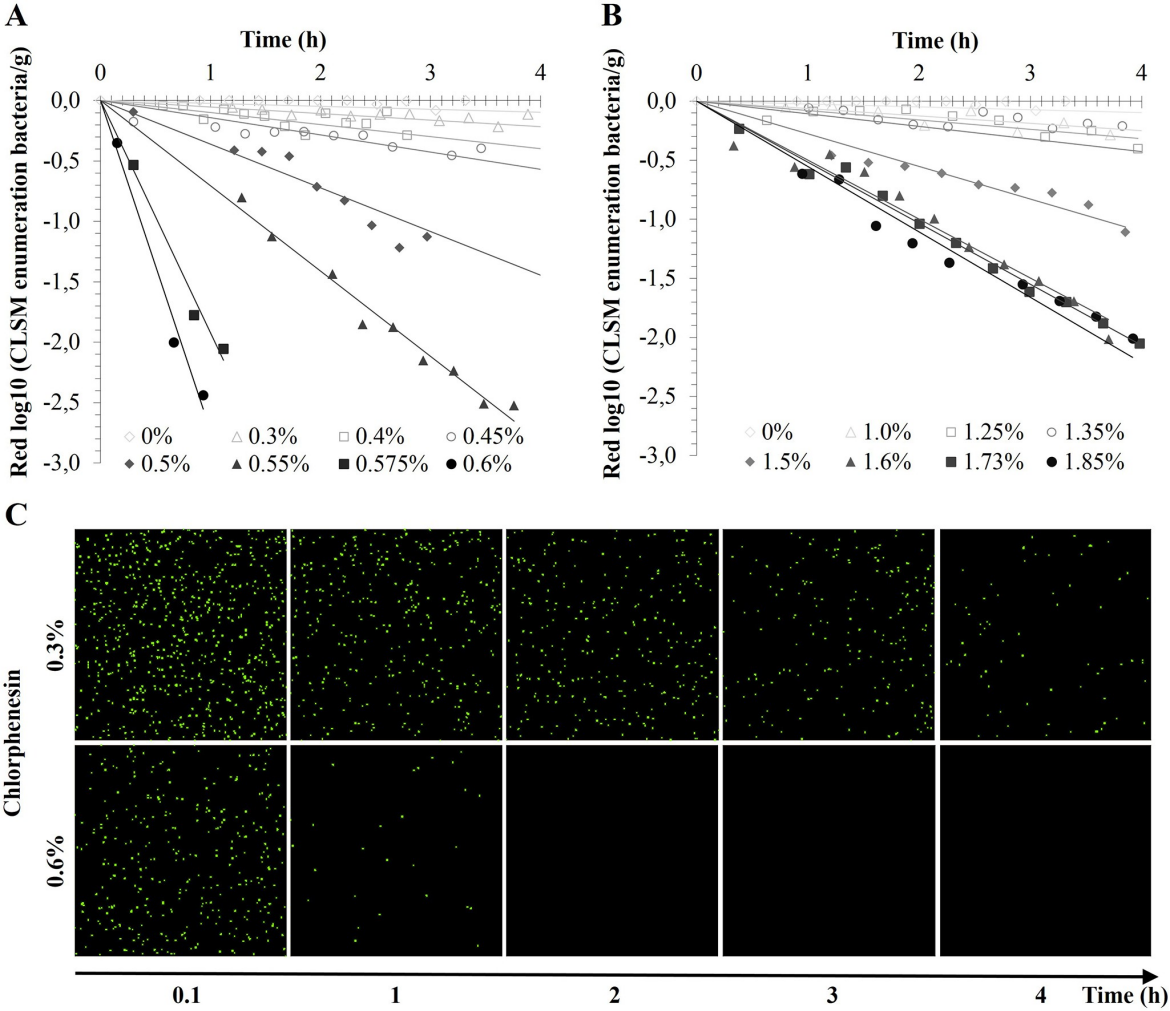
*S*. *aureus* inactivation kinetics obtained by HCS-CLSM in cosmetic model matrices with several concentrations of chlorphenesin (A) and benzyl alcohol (B). Example of the loss of bacterial fluorescence assessed by HCS-CLSM over time for two concentrations of chlorphenesin (C).

Fig 2 shows the kinetics of bacterial reduction obtained by CLSM during four hours for seven different concentrations of chlorphenesin (Fig 2A) or benzyl alcohol (Fig 2B). According to the correlation between enumeration by CLSM and plate counting (Fig 1), we only took into account data in the range of population above 6.10^6^ (maximum 2.5 log_10_ reductions). The higher the concentration of preservative the higher the slope of inactivation and the lower the Dc. For chlorphenesin, 0.3% is the smallest concentration for which Dc is measurable (17.89 h ± 1.12) on a CLSM kinetics (maximum 17h). For the range between 0.40 and 0.50%, Dc varies between 10.05 h ± 0.44 and 3.55 h ± 1.04. For the range between 0.55 to 0.60%, Dc varies between 1.48 h ± 0.10 and 0.45 h ± 0.07. For benzyl alcohol, Dc for the smallest concentration 1% is 28.09 h ± 7.50. From 1.5% Dc increasingly decreases to reach 1.07 h ± 0.05 at 1.85%. To obtain similar log reductions of *S*. *aureus*, the concentrations of benzyl alcohol should be higher than those of chlorphenesin. For example, we obtained one log reduction in 0.45 h ± 0.07 with 0.6% chlorphenesin whereas 1.07 h ± 0.05 is necessary with 1.85% benzyl alcohol. Fig 2C illustrates the loss of fluorescence of *S*. *aureus* in a model matrix with 0.3% and 0.6% chlorphenesin over time. With 0.3% chlorphenesin, the number of fluorescent bacteria decreases very slowly over time. At 4 h, the slight decrease of fluorescent bacteria number corresponds to a bacterial reduction of about 0.2 log bacteria/g (Fig 2A). In contrast, with 0.6% chlorphenesin, the fluorescent bacteria number decreased rapidly in 1h which corresponds to a reduction of 5.10^2^ bacteria/g (Fig 2A). After 2h, no bacteria were visible anymore.

### Estimation of Dc value according to the preservative concentration

**Fig 3:**
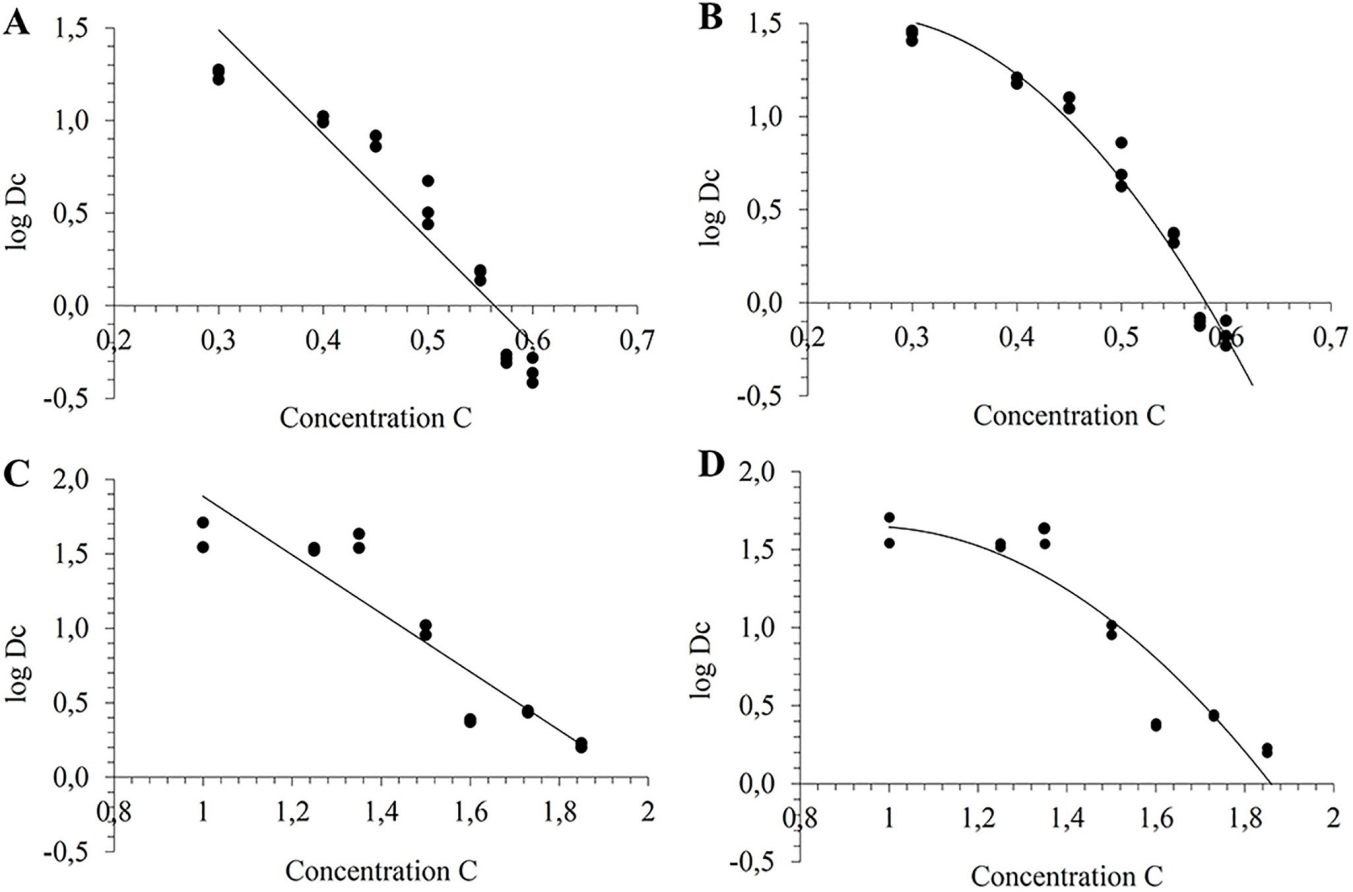
Relation between the Dc value and the concentration of chlorphenesin (A, B) and benzyl alcohol (C, D) by fitting of model#1 (A and C) and model#2 (B and D).

Semi-log models were used to fit datasets of Dc values upon the preservative concentration. The shape parameter *n* was set at 1 in model #1 (linear-model, Fig 3A and 3C) and set at 2 in model#2 (second-degree model, Fig 3B and 3D). Model parameters of the two models, Dc* and z_c_, are given in Table 1 together with the RSS and BIC for both preservatives. Second-degree model allows the lowest BIC for both preservatives, meaning that the shape parameter is significant. Accordingly to the BIC, model #1 does not fit well and was not used for the following prediction.

**Table 1:** Estimated parameters (and their 95% CI intervals) and performance criteria of both secondary models.

	Chlorphenesin		Benzyl alcohol	
	*model#1*	*model#2*	*model#1*	*model#2*
**n**	1	2	1	2
**Number of data**	21	21	14	14
C*	0.25	0.25	0.95	0.95
log (Dc*)	1.96 [1.76–2.16]	1.54 [1.47–1.62]	1.98 [1.70–2.22]	1.65 [1.44–1.78]
***z***_***c***_	0.18 [0.15–0.21]	0.27 [0.26–0.28]	0.51 [0.41–0.65]	0.71 [0.64–0.79]
***RSS***	0.76	0.20	0.72	0.60
***BIC***	-63.49	-91.48	-36.22	-38.86

### Prediction of bacterial log reduction on long periods

**Fig 4:**
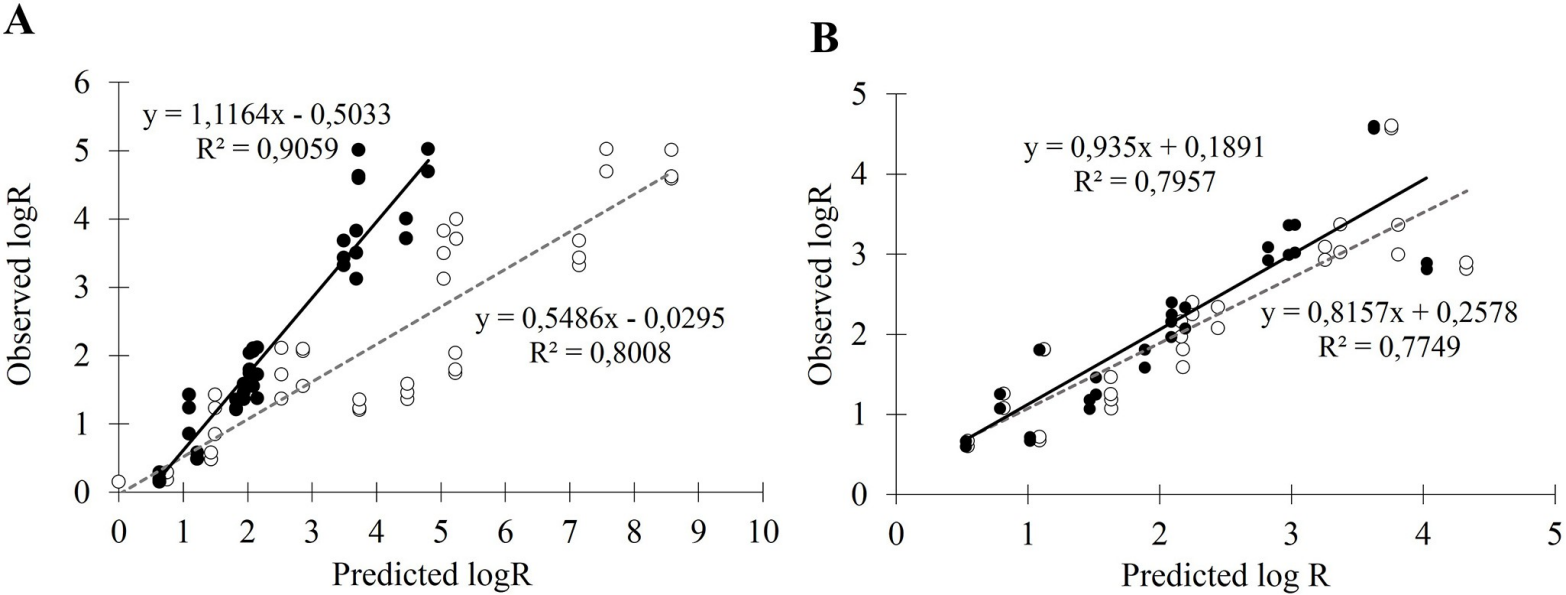
Correlation between the observed bacterial log-reductions and the predicted ones using Bigelow linear-model (white dots) or IQ model (black dots) for chlorphenesin (A) and benzyl alcohol (B).

**Fig 5:**
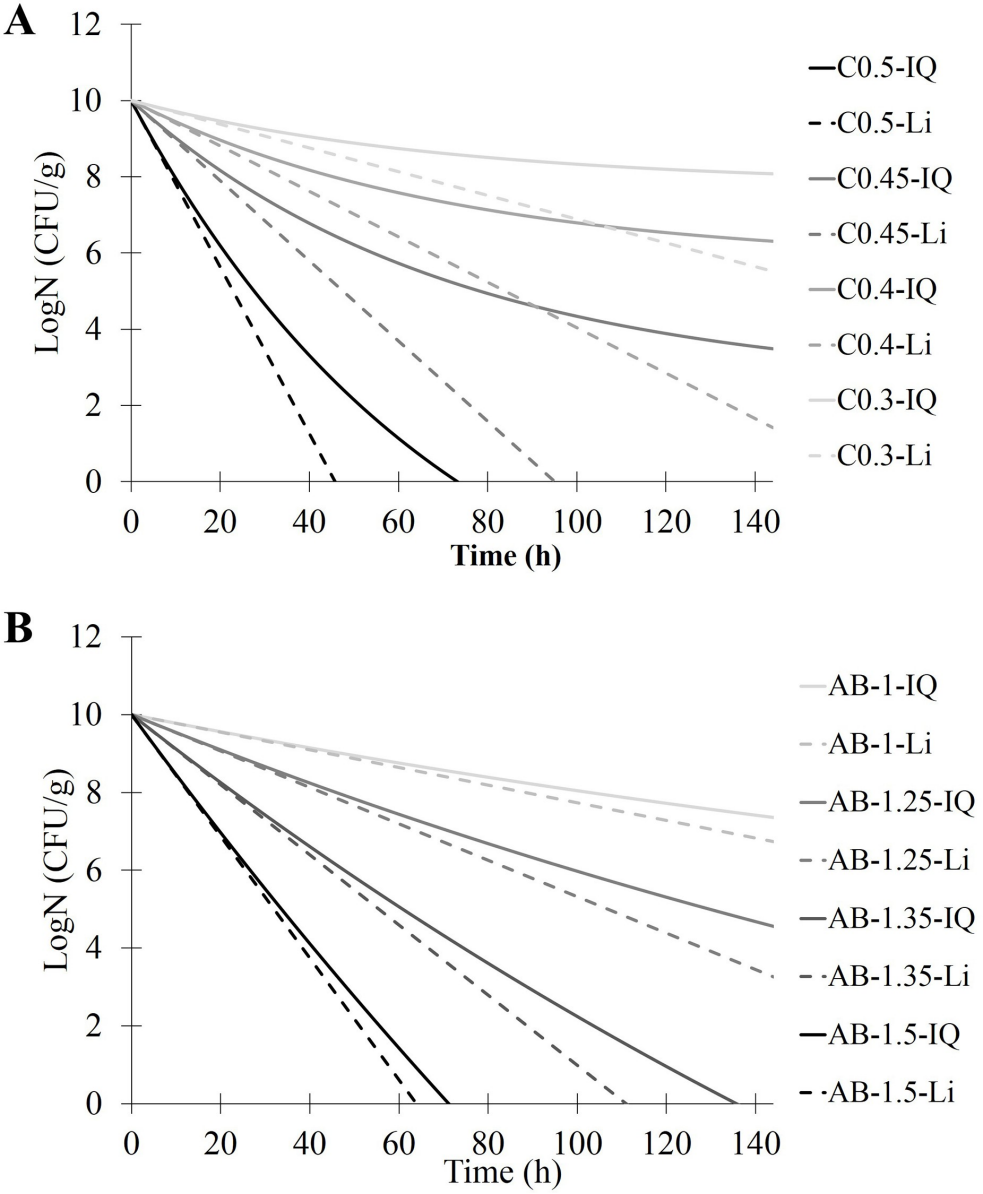
Illustration of the possible prediction of the evolution of the bacterial population over seven days for four concentrations of chlorphenesin (A) or benzyl alcohol (B) with Bigelow linear-model (dotted lines) or IQ model (plain lines).

Dc values at specific concentrations were first estimated from model #2. The logarithm reduction of the bacterial population was then calculated for specific times (from 1 to 7 days) using the Bigelow linear-model (Eq 1) or the IQ model (Eq 4). Fig 4 presents the relationships between predicted and experimental bacterial reductions. For both preservatives, the best combination is obtained when using IQ model for log-reduction estimation. For chlorphenesin (Fig 4A), the IQ model prediction for log-reduction datasets is far better than the linear model. The Q coefficient could be optimized at 0.0141 (CI 95% 0.0124–0.0156) and the slope of regression curve is 1.12 (R^2^ = 0.906). Linear model is less relevant with a slope around 0.55 and lower R^2^. For benzyl alcohol (Fig 4B), predictions with both models are less different than for chlorphenesin. The Q coefficient is optimized at 0.0043 (CI 95% 0.0022–0.0076) and the slope of the regression curve with the IQ model is 0.93 (R^2^ = 0.796). For both preservatives, one could note that the prediction is relevant only for maximum 5 log-reductions because of the initial level of contamination and the experimental protocol used to obtain the observed datasets. Fig 5 shows the prediction of the evolution of bacterial enumerations over seven days for 4 tested concentrations of chlorphenesin (Fig 5A) and benzyl alcohol (Fig 5B) with Bigelow linear-model (dotted lines) or IQ model (plain lines). These curves could be generated for any concentrations in the range of 0.3 to 0.6% of chlorphenesin and 1 to 1.9% for alcohol benzyl from model #2 and IQ model with respective Q to 0.014 for chlorphenesin and 0.003 for benzyl alcohol.

## Discussion

Challenge-tests are necessary to assess the efficiency of preservation in cosmetic products. Nevertheless, the procedure of the challenge-test is time-consuming due to the numerous enumerations by plate-counting necessary and to the results that are available only 48h after the last assessment point (day 28). By consequence, the challenge-test method lacks reactivity and flexibility for optimizing the preservation of a formula. In this study, we propose a new alternative method allowing the prediction of the log reduction of a bacterial population in long-term preservation by acquiring data on short-time periods. This method relies on the acquisition of CLSM kinetics of bacterial inactivation in the presence of several concentrations of preservatives during short times: acquisition during 4h is generally enough to evaluate Dc but 13h could be necessary for very low concentrations. Bacteria are first stained with a viability fluorescent marker, calcein-AM. This marker is widely used to assess bacterial viability by CLSM or by flow cytometry [31, 42]. Its precursor diffuses passively into the cytoplasm, where it is cleaved by intracellular esterases into green-fluorescent calcein [43]. This non-permeant fluorescent dye is released out of the cell when the membrane is permeabilized (dead cell).

We have first shown that the enumeration obtained by CSLM and dedicated image analysis can be correlated to bacterial plate-counting during the action (10 min to 4h) of the preservative (chlorphenesin or benzyl alcohol at specific concentrations). Our detection threshold by CLSM imaging is 6.10^6^ bacteria/g which is lower than the one obtained by CLSM by Auty *et al*. [29] (1.10^8^ bacteria/ml). This is probably due to the observed surface which was enlarged to a mosaic of 100 CLSM images. Here, bacterial CLSM enumeration is always higher (between 0.5 and 1 log) than the enumeration by plate counting. Auty *et al*. [29] also compared enumeration by CLSM and plate counting before they assess the viability of human probiotic strains in dairy products. They used Live/Dead Baclight marker and also underlined an overestimation of the CLSM enumeration of about 1 log. They suggested that this might be due to the bacterial clumping on plates. Indeed, the accuracy of enumeration by plate counting is usually estimated in the range of 0.3 to 0.7 log [23, 44]. However, we can notice that the difference between both techniques increases when enumeration decreases. Lower enumerations correspond to bacterial populations that remain alive after action of the preservative. Among this persistent population, a high fraction of bacteria is under stress which could explain why this fraction could not have the ability to recover and grow on agar plates while it is still stained by the viability marker by CLSM [24].

We used CLSM enumeration technique to follow the action of two preservatives at different concentrations in model cosmetic matrices. The bacterial inactivation kinetics was assessed by acquiring the calcein-AM loss of fluorescence over a few hours. These acquisitions were only possible thanks to the HCS-module of the CLSM. The automated high content screening (HCS) system is an emerging software solution that allows a CLSM to acquire automatically high content images for analysis of numerous samples, thanks to an automatically xyz-positioning in multiple wells as a function of time [45]. Automatic movements from well to well allow to acquire images for the same sample over time while avoiding photobleaching by enlightening each well only once. Moreover, we can also investigate several preservative concentrations over the same time lapse.

The CLSM method used during this study is very well suited to evaluate the efficiency of preservatives that cause membrane permeabilization. Chlorphenesin is a phenol ether with a chlorine atom and it belongs to the class of organo-halogen organic compounds. Phenols disrupt the cytoplasmic membrane and induce leakage of potassium ions of the cytosol. Their halogenation is known to improve their antibacterial activity [46]. Benzyl alcohol is an organic aromatic alcohol. Alcohols are known to damage cell membranes and denature bacterial proteins that are essential to the cell metabolism which leads to the cell lysis [46].

Chlorphenesin seems to be more effective than benzyl alcohol against *S*. *aureus* in the model cosmetic matrix. We observed that obtaining the same logarithm reduction needs lower concentrations of chlorphenesin than benzyl alcohol. According to the literature, the partition coefficient (logP) can be a parameter influencing bacterial inactivation [47]. The higher the logP the higher the antibacterial activity. Chlorphenesin could have a better ability than benzyl alcohol to intercalate into the bacterial membrane of *S*. *aureus* because its logP is higher (1.713) than the one of benzyl alcohol (1.100) [48, 49].

In this study, we were able to predict the number of log reduction at any time for one preservative at any concentration in a specific range from inactivation datasets obtained over short-term times. We fitted the datasets with two models describing the effect of the concentration on the log reduction time. These models derived from Mafart models [39] can take several forms by setting the shape parameter at 1 (linear model#1) or 2 (second-degree model#2). Mafart *et al*. (2001) compared these two first semi-log models for describing the effect of pH on the heat resistance of spores (reduction time D_T_) and showed that second-degree model presents a better safety than the linear one. From our side, we used the BIC calculation to choose the most relevant model while adjusting the minimum number of parameters. BICs of models#2 are better than model#1 for both chlorphenesin and benzyl alcohol (Table 1). This indicates that the preservative concentration and the contact time do not have a similar impact on the reduction time. As noticed by Mafart *et al*. (2001) for the effect of pH on the resistance of spores, we can hypothesize that the relationship between Dc and the preservative concentration is more complex than that of the effect of temperature on heat resistance. Hence, the linear model was discarded from the following prediction.

The next step was to predict, from the Dc values calculated with model#2, the log reduction of the bacterial population on longer times (up to seven days) using two primary models, the Bigelow linear model and the IQ model. The Q coefficient is the characteristic parameter of the IQ model which indicates the level of quenching of the preservative in the matrix (Lambert et al 2000) [41]. Below 0.005, which appears to be the case of benzyl alcohol, the level of quenching is very low and the inactivation curves are quite similar to linear log-survivor curves. On the contrary, the Q coefficient for chlorphenesin is 0.014 which indicates a quenching of the preservative in the matrix. The level of quenching increases over time as it is demonstrated by the comparison between the predictions of linear-model and IQ models (Fig 5). As it is not similar for both preservatives, we can hypothesize that it is influenced by the interactions between the antimicrobial and the matrix. As the model cosmetic matrix used here is an emulsion, we can hypothesize that chlorphenesin which has a higher log P (1.713) than benzyl alcohol (1.100) could progressively partition into the hydrophobic droplets, thus losing its preservative efficiency. Pernin et *al*. (2019) studied the antimicrobial activity of two natural phenolic compounds, ferulic acid and eugenol, against *Listeria monocytogenes* in a model oil-in-water emulsion. They showed that eugenol, which has the highest logP, loses its antibacterial efficacy in emulsified systems, in contrast of ferulic acid. The authors suggest that once in the emulsion, the more hydrophobic antimicrobial agent would preferentially partition in the lipid droplets and thus the remaining concentration in the aqueous phase would not be able to inhibit microorganisms [50]. Polarity, antimicrobial charge, and environmental conditions such as temperature, ionic strength, and pH can also play a major role in the effectiveness of an antimicrobial [51]. Electrostatic and hydrophobic interactions between antimicrobials and the matrix constituents, such as lipids, proteins and charged polysaccharides, could interfere with the antimicrobial activity [51]. For example, the addition of bovine meat proteins decreases the antimicrobial activity of phenolic compounds [52, 53]. Some gelling agents, such as hydroxypropylmethylcellulose, may be associated with the loss of effectiveness of preservatives [54]. Emulsifiers could also participate in the reduction of antimicrobial activity by sequestering antimicrobial molecules in micelles [50, 55, 56].

Nevertheless, from the estimations of Dc with model#2 and then of the log-survivors from IQ model, we propose here a method of prediction of the efficiency of two preservatives. The log-reduction of *S*. *aureus* population could be estimated at any concentration and after any time in a period of a few days for both tested preservatives.

This prediction is matrix- and preservative- dependent. The Q parameter is a characteristic of the interactions between them. This method should be challenged for many other couples of preservatives and matrices before it can be used for industrial prediction purposes. Moreover, some other microorganisms should be tested besides *S*. *aureus*, i.e. environmental strains isolated from contaminated cosmetic products. Calcein-AM is relevant for many bacteria including some Gram negative ones such as *Salmonella* [57]. However, it doesn’t work for some species including *Escherichia coli* [58]. Indeed, some strains, such as *Pseudomonas aeruginosa*, have efflux pumps that release the fluorescence outside the alive bacteria and prevent cell visualization [59]. To limit these pump interferences, it was suggested to add sodium azide in the staining solution [59], as used for the observation of biofilms [32]. Unfortunately, we cannot add this molecule in cosmetic matrices because it could modify the structure and composition of the formula. Hence, other impermeant fluorescent dyes should be evaluated.

## Conclusions

In this paper, we propose a rapid HCS-CLSM method associated with modeling to predict the preservative efficacy in a cosmetic matrix. This method could provide a quick evaluation of preservative efficiency and save a lot of time by replacing many microbiological analyses. It could be beneficially used for screening preservatives or for optimizing the formulation of a cosmetic product. Nevertheless this model has to be challenged in the future and adapted for several bacterial species, preservatives and matrices.

## Supporting information

S1 Data (XLSX)

S2 Data (XLSX)

S3 Data (XLSX)

S4 Data (XLSX)

S5 Data (XLSX)
